# Exploring the scope and applications of anti-doping measures in ultramarathon: an analysis of the positions of ultramarathon race organizers

**DOI:** 10.3389/fspor.2024.1406638

**Published:** 2024-05-22

**Authors:** Jill Colangelo, Alexander Smith, Stefanie Hachen, Michael Liebrenz

**Affiliations:** Department of Forensic Psychiatry, University of Bern, Bern, Switzerland

**Keywords:** ultramarathon, anti-doping policy, performance enhancing drugs, race organization, sport culture

## Abstract

**Introduction:**

With ultramarathon attracting burgeoning interest, evidence has emerged about doping behaviors. However, currently, research into the anti-doping policy landscape and the adoption of testing and athlete surveillance is limited, including the applicability of rubric from the World Anti-Doping Agency (WADA) and National Anti-Doping Organizations (NADOs). Consequently, it remains unclear if anti-doping provisions have been developed and enforced in ultramarathon, which is a timely consideration given growth in the sport.

**Methods:**

This study gathered perspectives on anti-doping and testing procedures from ultramarathon race organizers (UMROs). To that end, a sample of *n* = 35 prominent competitions was compiled using web materials and community engagement, encompassing elite and amateur entrants, diverse course designs, and prize money opportunities. Data-gathering was conducted across two phases between November and December 2023, with an initial review of UMRO web resources. Subsequently, UMROs were contacted via email to validate or ascertain their anti-doping and testing policies. Insights from UMRO respondents were reviewed and coded. UMROs who did not reply were excluded from the analysis.

**Results:**

Based on this methodology, the positions of *n* = 17 UMROs were captured, covering 159 ultramarathon races and approximately 96,500 annual participants. Of these, *n* = 8 UMROs did not have a self-developed policy and their rubric was pursuant to external authorities like WADA and NADOs. *n* = 4 had created a specific proprietary policy, which often incorporated WADO or NADO materials. The remaining *n* = 5 UMROs reported no anti-doping controls were in place at the time of the study. There was also notable heterogeneity in testing and surveillance, ranging from rigorous procedures to an absence of protocols. Interestingly, none of the included UMROs explicitly reported that they had enacted anti-doping measures against athletes.

**Discussion:**

Various determinants could inform these regulatory inconsistencies across UMROs, such as financial constraints, infrastructural and logistical barriers, cultural factors, and the lack of a unifying international federation in ultramarathon. Given the disparate approaches identified in our results, greater cooperation and education may be necessary to enhance understanding about the implications of doping and advance cohesive frameworks. This should involve collaborations with WADA and NADOs to promote best-practices and evidence-based exchanges within the community.

## 1 Introduction

### 1.1 Doping behaviors and performance enhancing drugs

Performance enhancing drugs (PEDs) and doping remain persistent issues in amateur and high-performance sports (1).^1^ Prominent cases continually emerge, leading some commentators to describe doping as an “epidemic” (2). Given the sensitivities around PEDs and doping, verifiable use rates are challenging to ascertain, particularly in amateur contexts where cases are more likely to go undetected. Nonetheless, estimates indicate that around 14%–39% of elite-level athletes may intentionally use PEDs (3). Separately, a meta-analysis across all levels of competition found that the prevalence of doping ranges from 0% to 73%, with the majority falling under 5% (1). Elite and amateur athletes may also unintentionally engage in doping, conceivably increasing prohibited substance use (4). Doping can be underpinned by heterogenous motivations, with banned substances entailing physiological and performance-based advantages, such as accelerated recovery, muscle mass growth, improved body leanness, and increased endurance (5). Other determinants include recreational use trends, socioenvironmental risk factors, and the consumption of PEDs for social and psychological goals (6, 7).

High-performance and amateur athletes may use PEDs like androgenic-anabolic steroids (AAS), hormones, stimulants, and analgesics (5, 8). Substance types can vary contingent on sport-specific pressures and demands (7). For instance, AAS have elevated use rates in sports like weightlifting, stimulants and hormones can be advantageous in cycling, and masking agents and diuretics may be more typical in different weight-sensitive disciplines (9). Despite this, doping (especially unsupervised use or combined substance use) can engender deleterious health outcomes (10). Notably, stimulants can heighten vulnerabilities for heat illness and cardiac dysfunction and non-steroidal anti-inflammatory drugs (NSAID) can be detrimental in high-intensity events (3, 8). Likewise, the potential implications of cannabinoids in sporting frameworks are not fully established (11).

### 1.2 Anti-doping governance

Internationally, the World Anti-Doping Agency (WADA) is an independent authority that seeks to “develop, harmonize and coordinate anti-doping rules and policies across all sports and countries” (12). WADA compiles an annual banned substances list, manages the World Anti-Doping Code, and promotes research and training, among other activities (12). To uphold consistent standards, WADA relies on National Anti-Doping Organizations (NADOs), such as UK Anti-Doping and the US Anti-Doping Agency, to function as the authority in a given country (13). Specifically, NADOs are tasked with carrying out testing, managing samples, investigating potential violations, and disseminating educational materials (13). In countries where there is no NADO, National Olympic Committees (NOCs) may fulfill these responsibilities. WADA also allows for Therapeutic Use Exemptions if athletes are able to provide comprehensive medical documentation to justify the need for a prohibited substance or method for treatment purposes (14).

Individual sports can also have a bespoke International Federation (IF) to implement WADA-informed anti-doping controls, which are typically aligned with the NOC (14). For example, the Union Cycliste Internationale (UCI) oversees all organized cycling events (15). This IF is a WADA signatory and although they utilize separate agencies to monitor and test riders and may implement additional regulations, the UCI broadly follows WADA guidelines (16). Within wider discipline-specific organizations like the UCI, national-level federations play a crucial role in attuning and implementing international guidelines across local sporting contexts (17).

WADA rules enforce athlete testing and surveillance inside or outside of competition (i.e., during training) (18). Conventional protocols require athletes to report to a Doping Control Station, provide a biological sample, and complete a Doping Control Form before analysis. Athletes bear “strict liability” for prohibited substances and doping transgressions often involve robust sanctions, such as competitive bans (12). Previous studies involving athletes have suggested support for WADA as upholding principles of fair competition (19). Yet, the validity of testing regimes and associated financial costs can be a concern (20), as can the medicolegal relevance of WADA's code in jurisdictions that criminalize doping (21). The accuracy and legitimacy of various testing methods has provoked debate, as has the general efficacy of anti-doping guidelines in amateur domains (22, 23). Similarly, the scope and evidence-base behind WADA's banned substance list has also been scrutinized (20, 24).

### 1.3 Ultramarathon, PED, and sporting governance

Among wider running disciplines, ultramarathon is attracting burgeoning interest. Over the past decade, entrant numbers have risen by 345% and an estimated six hundred thousand people are actively involved with the sport worldwide (25). Broadly speaking, distances beyond the standard marathon are considered as ultramarathons (i.e., >42.2 km). Races encompass mountainous terrains, forest trails, measured tracks, asphalt roads, hilly or flat courses, and combinations thereof (26). Accordingly, ultramarathoners may have distinctive characteristics than athletes in different events; competitors tend to be older even within the cadre of elite and professional runners (25). Additionally, as opposed to other disciplines where significant training demands are more associated with elite participants, the nature of long-distance racing typically requires high volume preparation at slower paces (i.e., multiple hours of running) (27).

Despite the growth in ultramarathon, there has historically been a lack of scientific inquiry into this discipline, which is particularly pronounced for PED and doping issues. Most accounts of PED use and doping (or suspicion of these behaviors) tend to be media-based and anecdotal (28), meaning overall prevalence rates are challenging to estimate and insights into testing, athlete surveillance, and sanctions are difficult to obtain. That said, available evidence suggests that certain ultramarathon entrants are using substances prohibited by WADA, such as cannabinoids, narcotics, and stimulants (29). In the cases where there is a clear intent to use a prohibited substance, athletes have been motivated by various factors. Some commentators have cited financial gains through winnings, more efficient recovery from injuries, and increased power due to muscle growth (30). Recent discussions have highlighted factors more intrinsic to amateur athletes, such as personal achievement, anti-aging, and body composition improvements (23). Likewise, it is possible that similar motivations for doping exist in ultramarathon as have been noted in other sports, despite possible differences in both culture and training demands [e.g., (5–7)].

In the limited scholarly literature dedicated to this topic, researchers identified that 27% of participants in an ultramarathon had taken drugs before the event and 18% during the race to manage pain. In this investigation, NSAIDs were most frequently consumed substance (9.8%), alongside painkillers (6.7%) (31). Elsewhere, in a survey of *n *= 609 ultramarathoners, 8.4% affirmed that they had used substances banned by WADA in competition or training, reporting that narcotics, cannabinoids, and stimulants were the most commonly consumed drugs (29). This study highlighted correlations between the use of prohibited substances and higher rankings, which could be influenced by socioenvironmental and competition pressures (29). In another investigation involving biological samples of *n* = 412 male ultramarathoners, 16.3% contained substances banned by WADA, including opioids, diuretics, glucocorticoids, beta-2 agonists, cannabinoids, and stimulants (32). In contrast to the previous findings, the presence of a prohibited substance was not correlated with better performances (32). Though prize winnings were not traditionally substantial in ultramarathon, financial incentives have increased, with single-winner payouts currently exceeding twenty-six thousand dollars (33). Winning or placing well helps athletes gain sponsorship from brands and this can be an important incentive as the majority of professional ultramarathoners (∼88%) make less than fifty thousand dollars per year (34, 35).

Unlike other running disciplines, ultramarathon is not represented by a unifying IF to develop systematic policies. Although ultramarathon associations do exist [e.g., (36)] they lack the cohesive mandate of conventional governance bodies. Nevertheless, the three prestigious ultramarathon entities, the International Association of Ultrarunners (IAU), the American Trail Runner Association (ATRA), and the International Trail Running Association (ITRA), all have different statements on PED use and their own preferences for testing methodologies (22, 34, 37). Controversies regarding the specific testing methodologies utilized across UMRO persist, particularly in the use of health screening services in lieu of drug testing (22, 38). For example, the UCI oversees cycling globally across divergent classifications, demographics, genders, and distances, but ultramarathon race organizers (UMROs) are often private entities with their own agendas. Specifically, ultramarathon events may require UMROs to prioritize varying aspects of race execution, such as trail building, environmental management, crowd control, and first aid access (39).

Correspondingly, since some ultramarathons are arranged by not-for-profit or charitable bodies, financial resources are also variable (40). Equally, certain ultramarathon course designs are certified while others are not, creating potential discrepancies in the applicability of oversight and in performance records and equivalence (41); for instance, it can be problematic to compare a mountainous one-hundred-and-sixty-kilometer race with ten thousand meters of climbing (42) to events that may require entrants to cover the most distance in twenty-four hours around a flat track. Moreover, the unique conditions of ultramarathons and the absence of an IF can complicate definitions of PED (22, 29), akin to broader disputes over what constitutes unfair advantage (24).

In sum, these infrastructural, logistical, and cultural considerations may contribute to variable attitudes towards PED and anti-doping in the ultramarathon community. Given these dynamics, we sought to garner a larger understanding of the positions of UMROs around anti-doping and PED policies. These perspectives could help to identify policy and implementation gaps, enhance transparency, and promote collaborations between UMROs and other stakeholders to refine best-practices across the sport.

## 2 Methods

### 2.1 Sample selection and data gathering

For collating and reviewing insights into the scope and enforcement of anti-doping and testing policies in ultramarathon events from UMROs, we followed a previous methodology that investigated organizational perspectives on mental health initiatives from sporting bodies (17). To that end, in October 2023, members of the research team compiled a list of *n *= 35 international UMROs responsible for overseeing single-runner ultramarathon races. Without a dedicated IF or national-level federations, this sample selection was informed by insights from stakeholders in the ultramarathon community and from relevant web materials [e.g., (43)]. The sample design was intended to represent the most prominent ultramarathons worldwide, capturing races with elite and amateur entrants, diverse course designs, and prize money opportunities.^2^

Having collated these details of these UMROs, a multi-stage data collection process was conducted from 13th November 2023 to 22nd December 2023. In the first stage, we evaluated UMRO web platforms to identify pertinent literature about anti-doping regulations and testing procedures. Subsequently, to verify this information or where no apposite materials were displayed online, we engaged in correspondence with UMROs via publicly available email addresses.

This second data-gathering phase focused on three enquiries. Specifically, UMROs were requested to provide details about their anti-doping rules and if they had ever enacted the provisions of this policy on suspicion of PED use. Additionally, UMROs were asked for their insights into how athletes are selected for doping tests and the timing of these practices. Within this correspondence, UMROs were advised that the answers would be used for the purposes of a research project for publication in an academic journal. No financial incentives were offered for participation.

### 2.2 Data analysis

From the answers given by the UMROs in our sample, the replies were formally interpreted and coded by two members of the research team into specific classifications using an inductive approach based on the content of the responses. In cases of disagreement, a third member of the research team was consulted, and a consensus was reached. Per this procedure, UMRO responses about anti-doping guidelines were coded into the following three classifications: “No reported anti-doping policy at time of study”, “Bespoke/self-developed anti-doping policy”, “Anti-doping policy pursuant to external authority”, and “No response”.

For the purposes of this study, the “Bespoke/self-developed anti-doping policy” category refers to cases where UMROs instituted their own rubric for the use of PED and testing strategies. The “Anti-doping policy pursuant to external agency” group encompasses UMROs who had not created their own policy but stated that they follow the existing guidelines of an established anti-doping authority, such as WADA or an NADO. Finally, those who did not reply to the enquiries or did not provide sufficient information were coded as “No response”.

### 2.3 Ethical considerations

In this investigation, information was collected from openly-accessible sources or was provided willingly by included UMROs. While some human input was required to obtain responses, the primary aim of this study was to collect insights into the policies and strategies from an organizational perspective. This information was either available to the public or shared voluntarily by the UMROs and no personal or sensitive data were involved and therefore formal ethical approval was not sought. However, care was taken to uphold the accuracy and integrity throughout the data collection process and during the evaluation and coding of the results, particularly given the sensitivities around doping and PED use.

## 3 Results

From the total sample of *n* = 35 UMROs, we received replies from *n* = 19 (54%), with *n* = 16 UMROs classified as providing no response (46%). One UMRO stated that they would reply to our enquiries based on the condition of anonymity and were therefore not included in the study. Moreover, two respondents were part of the Ultra-Trail du Mont-Blanc (UTMB) entity and were therefore classified under this grouping.

Accordingly, organizational perspectives from *n* = 17 distinct UMROs are outlined in the results. As UMROs can be responsible for overseeing more than one event, this sample of respondents represented around 159 single-runner ultramarathon races in over 30 different countries, covering approximately 96,500 entrants, with distances ranging from fifty kilometers to four hundred and fifty kilometers. The classifications of anti-doping policies from UMROs included in the analysis are presented in Table 1.

**Table 1:** An overview of anti-doping policies and perspectives from *n* = 17 ultramarathon race organizers.

Ultramarathon race organizer	Race(s)	Location(s)	Anti-doping policy	Approximate number of annual entrants
Ultra-Trail du Mont-Blanc (44)	UTMB World Series Events (124 competitions)	Worldwide	Bespoke/self-developed	56,000
Comrades Marathon Association (45)	Comrades Marathon	South Africa	Pursuant to external authority/authorities	23,000
Arista Eventos (46)	Transgrancanaria	Spain	Pursuant to external authority/authorities	4,100
Sinister Sports (47)	Five Total Events	Canada	Pursuant to external authority/authorities	3,500
Club di Montana Do Funchal (48)	Madeira Island Ultra Trail	Portugal	No reported policy at time of study	3,300
World Ultra Corporation (49)	Ultra X Races (13 competitions)	Worldwide	Bespoke/self-developed	1,560
Aravaipa Running (50)	Javelina Jundred	USA	Pursuant to external authority/authorities	1,300
Valle d'Aosta Trailers (51)	Tor Race Series (4 competitions)	Italy	Pursuant to external authority/authorities	1,050
Vermont Adaptive (52)	Vermont 100 (2 competitions)	USA	Pursuant to external authority/authorities	900
Montaine Spine (53)	The Spine Race	UK	No reported policy at time of study	550
Ourea Events (54)	Dragon's Back Race	UK	No reported policy at time of study	500
West Highland Way Race (55)	West Highland Way Race	UK	Pursuant to external authority/authorities	300
Atlantide Organisation (56)	Marathon des Sables	Morocco	Bespoke/self-developed	250
Fatdog Management (57)	Fatdog 120	Canada	Pursuant to external authority/authorities	200
Hardrock Hundred Board of Directors (42)	Hardrock 100	USA	No reported policy at time of study	140
Western States Endurance Run Foundation (58)	Western States Endurance Run	USA	Bespoke/self-developed	140
Hurt Inc. (59)	Hurt 100	USA	No reported policy at time of study	135

Per the coding and interpretation of the findings from the data gathering process, *n* = 8 UMROs stated that they did not have a self-developed policy and their regulations were pursuant to external agencies (classified as “Anti-doping policy pursuant to external authority”). These UMROs followed the guidelines of a separate authority such as WADA and would therefore enforce policy through NADOs like the United States Anti-Doping Agency and Drug-Free Sport. In these *n* = 8 cases, UMROs were using the banned substances list of an external agency upon which to base its regulations.

Furthermore, *n* = 4 UMROs stated that they have a proprietary policy (“Bespoke/self-developed anti-doping policy”), which was created specifically for the ultramarathon events they manage. For clarity, it should be noted that the use of a proprietary anti-doping policy did not signify a rejection or rebuttal of external agencies and may use aspects of these codes or WADA's banned substance list. Finally, *n* = 5 UMROs indicated that they did not have an anti-doping policy at the time of our correspondence (i.e., “No reported anti-doping policy at time of study”). To capture the nuances in the positions and types of anti-doping policies and testing procedures from included UMROs, responses are described qualitatively below.

### 3.1 Anti-doping policy pursuant to external authority (*n* = 8 UMROs)

Within this category, *n* = 3 UMROs reported that they adhere to the guidelines of external authorities and conducted regular testing. For example, in South Africa, the UMRO for the Comrades Marathon, which is licensed by KwaZulu Natal Athletics, Athletics South Africa, and World Athletics and has approximately 23,000 entrants, complies with national and international anti-doping rules. This association affirmed that all top ten men and women are tested on race day and additional assessments can be requested dependent on the scenario. In their correspondence, this UMRO noted that they will strengthen their cooperation with Drug Free Sport (the South African anti-doping agency), which complies with WADA's list of banned substances, from 2024. This will entail collecting pre-race whereabouts for spot checks and arranging out-of-competition testing for local and international top contenders (as defined by past performances).

Similarly, the Valle d'Aosta Trailers, organizers of the Tor Series of races up to 450k, asserted that they adhere to WADA guidelines for anti-doping; testing in this event is performed by the Nuclei Antisofisticazioni e Sanità (NAS) of the Carabinieri, a law enforcement agency of the Italian government that is tasked with surveillance of athletic facilities and competitions (60). Likewise, in the Transgrancanaria race, the UMRO stated that they follow rubrics from established agencies (61); specifically, this UMRO confirmed that anti-doping testing has been conducted previously for individual athletes in collaboration with the Spanish NADO. They also confirmed that participants in the podium positions in this event are routinely tested.

The remaining *n* = 5 UMROs stated that they comply with external rubric for anti-doping but did not indicate that they conducted routine testing. In practice, this meant that they conduct the race in accordance with established anti-doping policies (e.g., following the WADA prohibited substance list), but do not/cannot test to confirm this assumption. As a certified event in Canada, Fat Dog 120 said that they follow procedures from BC Canada, World Athletics, and WADA. Moreover, this UMRO stated that if an entrant's results were in question or if any PED use was suspected, they would enact BC Athletics and WADA rubric, but they have not imposed this to-date. An UMRO for another ultramarathon race series with 3,500 total entrants in Canada, Sinister Sports, attested they follow WADA rules for prohibited substances and competitors have periodically requested therapeutic exemptions. Further, this UMRO said they would consider testing on a case-by-case basis, but this was not applied routinely due to financial and logistical barriers.

Separately, competitors in the Vermont 100 in the United States must follow USA Track & Field (USATF) rules for banned substances pursuant to WADA's list and anti-doping policy. This UMRO referred to the USATF list of banned athletes to determine an athlete's eligibility to participate but did not cite any testing procedures. The Javelina Jundred stated that though they do not have a self-developed anti-doping policy, they would test an athlete if a world record was achieved but have not yet tested any competitors. In the United Kingdom, the West Highland Way Race is licensed by Scottish Athletics and therefore follows UK Athletics' anti-doping controls. Additionally, this UMRO includes a statement in the race materials expressly prohibiting NSAID use; this entity also noted that they have not carried out anti-doping tests.

### 3.2 Bespoke/self-developed anti-doping policy (*n* = 4 UMROs)

The Marathon des Sables race in Morocco involving 250 participants over 250 kilometers has created a comprehensive set of guidelines covering PED and doping (56). The UMRO noted that these require all participants to provide information on medications used thirty days before the event, and any TUEs and ongoing anti-doping sanctions (56). In this event, entrants must abide by WADA's banned substance list and give samples on request thirty days before the competition or fifteen days after (56). Analogously, athletes are prohibited from consuming narcotics, NSAIDs, and cannabidiols twenty-four hours prior to the competition, general intravenous infusion or thyroid synthesis hormones seven days before the event, and intravenous iron infusion thirty days from the race (56). Breaching these rules can result in a warning or elimination from the Marathon des Sables (56).

The UTMB World Series Events, incorporating 124 ultramarathon races and approximately 56,000 runners over 30 countries, has general anti-doping resources for all its events UTMB [e.g., (62)]. Comparable to other UMROs, the guidelines from UTMB utilize the WADA proscribed substance list and alongside providing general advice about supplements, stipulate that all athletes may be subject to testing inside and outside of competition and that individual races in the UTMB World Series can enforce additional regulations (44).

Though the Western States Endurance Run has become a UTMB World Series Event, the UMRO consider the race to be wholly independent, i.e., governed and financed separately from any other entity (63). With 140 competitors, the UMRO for this race introduced its own anti-doping rubric in 2017 (63). This documentation uses WADA's banned substance list and is continually updated and subject to revision (63). The UMRO stated that policy at the Western States Endurance Run allows any male or female athlete to be tested before or after the event and in general, past tests have been conducted on top age group finishers or elite-level participants, but no runner has been sanctioned to-date.

Finally, the UMRO for Ultra X events, which has thirteen races and an estimated 1,560 participants, have published their own policy for anti-doping, which integrates materials from WADA and other bodies about banned substances and supplements (64). Nonetheless, the protocols of this regulation have not been enacted on suspicion of doping behaviors to-date and unless specific concerns are raised, this UMRO stated that routine testing is not carried out owing to financial constraints.

### 3.3 No reported anti-doping policy at the time of the study (*n* = 5 UMROs)

With a course covering over one hundred and sixty kilometers in the United States, the UMRO for the Hardrock 100 affirmed that they do not have a written anti-doping policy when the study was conducted. Consequently, no athlete has been subject to doping controls and any possible testing would be at the discretion of the medical director. When the research was carried out, the UMRO for the Madeira Island Ultra-Trail in Portugal had not yet developed a policy for the event in 2024 and was thereby classified in this category.

Likewise, Hurt 100 in the United States, which attracts approximately 135 entrants a year, has not developed an anti-doping regulations or testing measures due to limited financial resources. This UMRO cited cultural factors as determining this situation, such as the non-competitive nature of the race and a reliance on volunteers for organization. The UMRO for the Dragon's Back Race in the United Kingdom also reported that they had no formal anti-doping policy at the time of reply and was therefore incorporated into this grouping. This position was the same for the Spine Race in the United Kingdom.

## 4 Discussion

### 4.1 The landscape of anti-doping policies and testing procedures in ultramarathon

The results demonstrate that anti-doping controls and testing procedures in ultramarathon are not standardized or universally applied, irrespective of the course design or the country of the UMRO. From the responses, *n* = 12 UMROs reported on a self-developed policy or indicated that they followed regulations pursuant to external authorities, whereas at the time the research was conducted, the remaining UMROs (*n* = 5) noted that they had not yet introduced anti-doping guidelines. This heterogeneity may reflect broader complexities and challenges in the enforcement and monitoring of doping behaviors within the sport, as has been highlighted in the media and by academic researchers (28, 30).

Correspondingly, our findings show that UMROs rely on information and testing from a variety of sources contingent on the national framework, including WADA and other public agencies [e.g., (62)]. Interestingly, none of the UMROs explicitly stated that they had ever had to enact their anti-doping policy, suggesting either a low incidence of detected doping within the specific races included in our results or possible limitations in the effectiveness and enforcement and existing measures for UMROs who did note that they conducted testing. That said, these responses could also be influenced by the sensitivities and privacy surrounding PED use and anti-doping sanctions, which may have entailed a reluctance to disclose applicable details; this is particularly pertinent since doping behaviors have previously been found in ultramarathon (31). In this regard, the lack of enactment of policies appears to contrast with the aforementioned discussion of the increasing concerns around PED use and doping in ultramarathon. In the authors' opinion, this may point towards the inability of current systems to keep pace with need due to the high cost of testing, inconsistencies in protocol, and associated stigma, as is discussed in greater detail in Section 4.2.

While many UMROs with anti-doping policies integrated the WADA list of prohibited substances as a foundation of their regulations, there were explicit stipulations for certain drugs and supplements. This was demonstrated by organizational insights from the West Highland Way Race, which expressly referenced the prohibition of NSAIDs in their event guidance, and the Marathon des Sables that specifically cited restrictions on NSAIDs and other substances. Although the direct effects of NSAID use have been debated and it has had correlations with adverse events in endurance cyclists, similar observations have not been established in ultramarathon (65).

Equally, there appears to be limited consensus on the implementation of drug testing and surveillance; UMRO positions ranged from routine to selective testing practices, and in some cases, to an absence of testing altogether. Furthermore, our results revealed discrepancies in the agencies responsible for testing measures in ultramarathon, with UMROs highlighting this as the role of NADOs, race medical directors, and law enforcement. This finding may be expected as our analysis incorporated UMROs across various countries and responsibilities for doping tests can vary cross-jurisdictionally (13). Yet, inconsistencies in testing are not specific to ultramarathon and have been illustrated in wider doping literature across sporting disciplines (19). More generally, the integrity of several testing strategies have been debated but there remains a lack of scientific study on what protocols may be most sensitive, accurate, safe, and cost-effective for larger athlete groups (20, 66).

### 4.2 Reasons for the variability in anti-doping policies in ultramarathon

As demonstrated by the organizational perspectives presented in our results, it is likely that onerous financial considerations are a significant barrier to the creation and enforcement of anti-doping policies in ultramarathon. Typically, WADA-compliant testing is estimated to require over $230 million per year and individual testing can cost $600–$700 USD per athlete (67). This may be unaffordable for many UMROs. Notably, several UMROs, including those from Ultra X, the Canadian Death Race, and the Hurt 100, cited financial obstacles as an explanation for their lack of testing provisions. While some UMROs operate on a for-profit basis [e.g., (44)], many events are overseen by non-profit organizations or may even be created as fundraising events for charitable entities, all of which can have different agendas and priorities (52). Hence, it could be complex to implement policies that would incur equal financial burdens across races that do not have comparable budgetary models, particularly without the guidance of dedicated ultramarathon IF.

Logistical specificities in the discipline may present further impediments for cohesive anti-doping policies across ultramarathon. As previously discussed, there can be variations in the style, terrain, distance, rules, and distance of ultramarathon events. For example, certain races require entrants to run as many laps as possible on a flat, .4 kilometer track, but the Tor des Gèants covers 330 kilometers and has an overall elevation gain of 24,000 meters with a cut-off of 150 h (51). The former allows athletes to set up a station where they may eat, sleep, and take shelter. However, the Tor des Gèants involves long stretches of time alone in challenging terrain and unpredictable weather, necessitating survival gear like a blanket and a knife (51).

Given these aspects, it may be logical to assume that it would be unfeasible to apply overarching anti-doping rules across divergent competitions. Analogously, the logistics of these races may preclude testing and surveillance protocols; this was mentioned by the UMRO for Hurt 100, who highlighted the voluntary nature of race organization. Nevertheless, other sporting governance bodies and IFs have successfully unified various types of sport. This includes the UCI, which encompasses BMX, mountain biking, road cycling, and track cycling under its mandate, which take place on a range of courses and within disparate sporting frameworks (15). Likewise, sociocultural discrepancies can be significant among cycling disciplines, which also have the added complication of gender-specific competition, yet a single anti-doping policy stands across cycling (68).

Another reason for heterogenous anti-doping approaches in ultramarathon may conceivably stem from a reluctance to acknowledge that such regulations are necessary. In this regard, certain UMROs in our investigation accentuated the notion that entrants were amateurs or competing solely for recreational purposes, perhaps implying that ultramarathons are unconventional and perceived as less susceptible to doping than other sports. Ultramarathon can be characterized by unique sociocultural dynamics, emphasizing physical challenges and a sense of personal accomplishment (69). Authenticity plays a key role in the community and athletes strongly resonate with the concept of running for “the love of the sport” and enduring pain (66). However, there is anecdotal discussion of PED use in sports like rock climbing which, until recently, were also not generally considered to be mainstream (70). In the authors' opinion, increased interest, participation, investment, and media coverage in ultramarathon could require detailed consideration of factors like doping in ultramarathon, which may have conventionally associated with more mainstream sports.

That said, recent inquiries into the influence of nationality in ultrarunning suggests that collegial, non-competitive perspectives may be more representative of North American athletes (71). Despite this, in the authors' opinion, it may still be difficult for community stakeholders to consider that athletes are participating due to alternative motivations or that ultramarathoners may knowingly consume banned substances even when prizes or prestige are not at stake. Additionally, the reported consumption of banned substances and the association between substance use and higher rankings, as previously identified, challenge this perception and underline a need for greater awareness (29). More generally, calls for vigilance within the community about the consumption of banned substances are especially timely as sophisticated techniques to evade positive doping results become increasingly common (20).

### 4.3 Community collaborations and education in ultramarathon

With its burgeoning popularity, ultramarathon is currently at an important point in its history where external pressures and socioenvironmental determinants may encourage the use of banned substances, akin to the trajectory of other sports [e.g., (5, 6)]. Presently, as reflected in our findings, there are divergent positions about the scope of anti-doping policies in ultramarathon and who is responsible for implementing these measures and testing athletes. Accordingly, there is a need for best-practices and cohesive approaches to be promoted across the sport. Yet, owing to infrastructural constraints, financial obligations, and the lack of a bespoke IF, it remains difficult to see how this can be coordinated. Elsewhere, recent commercial developments in ultramarathon have caused controversy and undermined the notion of there being a unified ethos across the community (72).

To harmonize positions within the sport bidirectional and cross-cultural exchanges within the sport are necessary to promote best practices and standardize approaches. Within this context, constructive dialogues in ultramarathon could yield progressive benefits, informed by collaborations between UMROs, athletes, and other stakeholder groups. For optimizing their reach, these should include governing bodies of related sports and established anti-doping authorities, including WADA and NADOs. In lieu of a dedicated ultramarathon IF, prominent associations like IAU, ITRA, and ATRA can create a platform for these conversations, bringing together disparate organizational priorities and cultural nuances. Moreover, as banned substance lists are continually updated, periodic knowledge exchanges involving UMROs, coaches, and athletes should be emphasized. Relatedly, additional research into PED use in ultramarathon is essential to underpin these discussions. Studies have illustrated consumption rates of prohibited substances in specific events (20, 29, 63) and more work is necessary to better understand the health implications of PED in ultra-distance events to inform evidence-based recommendations.

Correspondingly, we also advocate for greater educational provisions for athletes and coaches in the ultramarathon community. Given that ultra-endurance athletes may rely on data-driven training plans for race preparation, presenting this audience with evidence-based information could accentuate the health risks associated with the use of banned substances. In this regard, WADA has an extensive plan for training, utilizing a range of strategies for outreach. However, it is unclear which, if any, ultramarathon athletes and coaches have been involved in these initiatives (73). At an individual race level, we suggest that UMRO publicize a clear statement on anti-doping policy upon race sign-up and apposite information on race websites. Notably, several UMROs in our analysis had publicly-available resources on doping and this should be encouraged as a best-practice throughout the sport (63). For larger races, informational programs can be held to educate entrants on the sociolegal consequences of doping, together with the possible dangers to their health and safety stemming from PED usage.

## 5 Limitations and future research directions

This investigation provides an overview of the heterogeneity of anti-doping controls and testing measures in ultramarathon by collecting and evaluating organizational perspectives from a range of international UMROs. Yet, the methodology has several limitations that could be considered in future research.

Firstly, we aimed to collate information from the most prominent ultramarathons in the sport, which would encompass elite and amateur runners, opportunities for prize money, and a large number of finishers. In the absence of a singular IF, we developed the list of UMROs through web resources and stakeholder engagement within the ultramarathon community. As this was a self-selected sample with a non-systematic approach, this may raise relevant concerns about bias or mean that more specific races or athlete groups were overlooked in the analysis. However, given the nature of this study in a relatively unexplored field, our approach yielded preliminary insights, rendering it a valuable basis for understanding the broader landscape.

Given that the responses from UMROs were presented through written commentary, two members of the research team interpreted and coded these answers. This may have led to issues of subjectivity or reproducibility, which cannot be completely discounted. That said, to uphold accuracy and reach consensus in the interpretations, a third member of the research team was consulted in cases of disagreement. Moreover, as this study adopted a cross-sectional design, it is possible that anti-doping or testing guidelines have been newly-introduced by UMROs or have been recently updated.

No financial incentives were offered for the UMROs to be included in this project, and all were informed about the scope of the research and that it was intended to be published in academic journal. Consequently, *n* = 1 UMRO asked to not be named, which precluded them from participating. This lack of anonymity may have prevented other UMROs from engaging into correspondence. Additionally, conducting the data-gathering in English may have led to misunderstandings and incomplete or inaccurate replies, especially since certain UMROs in the sample were from non-English language countries; resultantly, this may also have conceivably limited the inclusion rate.

Our results included responses from *n* = 17 (48%) of the *n* = 35 UMROs identified in the sample selection, meaning some events selected in the initial sampling are not represented. Nevertheless, we believe that our findings from 17 UMROs covering 159 races and an estimated 96,500 competitors from over 30 countries offers preliminary and diverse perspectives into anti-doping regulations and testing protocols throughout the sport. These can provide a basis for future research directions around doping in ultramarathons.

For example, a comparative analysis between ultramarathons and other endurance sports might reveal best practices and regulatory gaps, offering greater insights into the potential for unified anti-doping strategies. Analogously, as there is scant evidence about stakeholder impressions on the efficacy and value of testing regimes in ultramarathon, this could be incorporated into a detailed qualitative investigation. Finally, inquiries into the levels of awareness about the effects of PEDs among ultramarathon athletes and coaches could be beneficial for informing tailored outreach and education within the community to promote better health outcomes.

## 6 Conclusions

The results from this study underline the diversity and associated complexities of anti-doping policies and testing provisions in the ultramarathon community. Our findings underline that approaches can significantly differ across races, regardless of terrain, country, and distance. Specifically, policies ranged from being pursuant to those developed by external authorities, through to bespoke documentation, and an absence of protocols. Likewise, testing was conducted routinely, inconsistently, or not at all. According to the included UMROs, no organization expressly reported that they had enacted the provisions of their policies against any athletes. Several UMROs suggested that the lack of an anti-doping policy or their reasons for not enforcing these regulations was determined by various factors, including financial concerns, limited prize money opportunities, and sociocultural attitudes that attenuated the possibility of PED use.

With the growing popularity of ultramarathon, it is important to note that a reluctance to acknowledge the implications of PED and doping in the community does not nullify the existence of these behaviors. Increased interest, participation, investment, and media coverage in the sport may thus require collaborations between stakeholders to develop coherent best-practices. In doing so, the ultramarathon community can better safeguard the spirit of competition and the ethos of ultra endurance sports.

## Data Availability

The original contributions presented in the study are included in the article/**Supplementary Material**, further inquiries can be directed to the corresponding author.

## Footnotes

^1^As both legal and illegal substances may be categorized as PEDs, it is important to note that while athletes using PEDs may be in violation of anti-doping rules, they may not be engaging in criminal activity contingent on the jurisdiction (2). Specifically, in certain countries, the use of any PED in competition, irrespective of its legal status, is a punishable offense (2). For the purposes of this discussion, any drug that is in violation of anti-doping policies shall be described as “prohibited” or “banned”, though it may not be illegal to possess or use the substance in non-sporting contexts. Likewise, the term “doping” refers to the use of a prohibited substance in violation of established anti-doping rules, notwithstanding its legality in judicial contexts.

^2^Details of the full sample of n=35 UMROs are presented in the Supplemental Materials.
