# Supplementary material for: Exploring the scope and applications of anti-doping measures in ultramarathon: an analysis of the positions of ultramarathon race organizers
Source: Front Sports Act Living. 2024 May 22;6:1406638. doi: 10.3389/fspor.2024.1406638 (PMC11150625; doi:10.3389/fspor.2024.1406638)
Supplement: Supplementary file 1 [file Datasheet1.pdf]

Supplemental Materials

Full sample details of races to be included in the study.

Race	UMRO	Web resources
Vermont 100	Vermont Adaptive	https://vermont100.com/
TOR330 - Tor des Géants	Valle d'Aosta Trailers	https://www.torxtrail.com/
Transalpine Run	Plan B Event Company	https://www.transalpine-run.com/
Leadville Trail 100 Run	Life Time Inc.	https://www.leadvilleraceseries.com/run/leadvilletrail100run/
Hurt 100	Hurt Inc.	https://hurt100.com/
UTMB	UTMB	https://utmb.world/
Madeira Island Ultra-Trail	Club di Montanha Do Funchal	https://www.miutmadeira.com/
Ultra Tour Monte Rosa	KORA Explore	https://www.ultratourmonterosa.com/
The Spine Race	Montaine Spine	https://www.thespinerace.com/
Dragon's Back Race	Ourea Events	https://www.dragonsbackrace.com/
Marathon Des Sables	Atlantide Organisation	https://www.marathondessables.com/
Grand Raid De La Réunion	Association Le Grand Raid	https://www.grandraid-reunion.com/
West Highland Way Race	West Highland Way Race	https://westhighlandwayrace.org/
Comrades Marathon	Comrades Marathon Association	https://www.comrades.com/
Badwater Races	Adventurecorps Inc.	https://www.badwater.com/
Lavaredo Ultra Trail 50K, 80K, 120K	UTMB	https://lavaredo.utmb.world/
Spartathlon Ultra Race	Spartathalon	https://www.spartathlon.gr/en/home/
Ultra X Races	World Ultra Corporation	https://ultra-x.co/

Extreme Adventure Races (Fire & Ice, Viking, Annapurna)	Extreme Adventure Races	https://www.extremeadventureraces.com/
Tarawera Ultramarathon	UTMB	https://tarawera.utmb.world/
Montane Lapland Arctic Ultra	The Great Outdoors	https://lapland.arcticultra.de/
Falklands Ultra	Combat Stress	https://combatstress.org.uk/support-us/falklands-ultra
Transgrancanaria	Arista Eventos	https://transgrancanaria.net/en/
Trans Atlas Marathon	Ahansal Events	https://www.transatlasmarathon.net/
Javelina Jundred	Aravaipa Running	https://aravaiparunning.com/network/javelinajundred/
Desert RATS Kokopelli	Gemini Adventures	https://geminiadventures.com/run/desert-rats-150
Trail Verbier St-Bernard	UTMB	https://verbier.utmb.world/
Grand 2 Grand Ultra	Grand 2 Grand Ultra	https://g2gultra.com/
Fat Dog 120	Fatdog Management	https://www.fatdog120.ca/
Gorge Waterfalls 50K, 100K	Daybreak Racing	https://www.daybreakracing.com/gorge-waterfalls
Squamish 50	Coast Mountain Trail Running	https://squamish50.com/
Canadian Death Race	Sinister Sports	https://www.sinistersports.ca/
Haliburton Forest Races	Haliburton Forest Trail Ultra	https://www.haliburtonforest100.org/
Hardrock 100	Hardrock Hundred Board of Directors	https://hardrock100.com/
