# Supplementary material for: Exploring the scope and applications of anti-doping measures in ultramarathon: an analysis of the positions of ultramarathon race organizers
Source: Front Sports Act Living. 2024 May 22;6:1406638. doi: 10.3389/fspor.2024.1406638 (PMC11150625; doi:10.3389/fspor.2024.1406638)
Supplement: Supplementary file 2 [file Table1.docx]

**Supplemental Materials**

Full sample details of races to be included in the study.

| **Race** | **UMRO** | **Web resources** |
| --- | --- | --- |
| Vermont 100 | Vermont Adaptive | https://vermont100.com/ |
| TOR330 - Tor des Géants | Valle d'Aosta Trailers | https://www.torxtrail.com/ |
| Transalpine Run | Plan B Event Company | https://www.transalpine-run.com/ |
| Leadville Trail 100 Run | Life Time Inc. | https://www.leadvilleraceseries.com/run/leadvilletrail100run/ |
| Hurt 100 | Hurt Inc. | https://hurt100.com/ |
| UTMB | UTMB | https://utmb.world/ |
| Madeira Island Ultra-Trail | Club di Montanha Do Funchal | https://www.miutmadeira.com/ |
| Ultra Tour Monte Rosa | KORA Explore | https://www.ultratourmonterosa.com/ |
| The Spine Race | Montaine Spine | https://www.thespinerace.com/ |
| Dragon's Back Race | Ourea Events | https://www.dragonsbackrace.com/ |
| Marathon Des Sables | Atlantide Organisation | https://www.marathondessables.com/ |
| Grand Raid De La Réunion | Association  Le Grand Raid | https://www.grandraid-reunion.com/ |
| West Highland Way Race | West Highland Way Race | https://westhighlandwayrace.org/ |
| Comrades Marathon | Comrades Marathon Association | https://www.comrades.com/ |
| Badwater Races | Adventurecorps Inc. | https://www.badwater.com/ |
| Lavaredo Ultra Trail 50K, 80K, 120K | UTMB | https://lavaredo.utmb.world/ |
| Spartathlon Ultra Race | Spartathalon | https://www.spartathlon.gr/en/home/ |
| Ultra X Races | World Ultra Corporation | https://ultra-x.co/ |
| Extreme Adventure Races (Fire & Ice, Viking, Annapurna) | Extreme Adventure Races | https://www.extremeadventureraces.com/ |
| Tarawera Ultramarathon | UTMB | https://tarawera.utmb.world/ |
| Montane Lapland Arctic Ultra | The Great Outdoors | https://lapland.arcticultra.de/ |
| Falklands Ultra | Combat Stress | https://combatstress.org.uk/support-us/falklands-ultra |
| Transgrancanaria | Arista Eventos | https://transgrancanaria.net/en/ |
| Trans Atlas Marathon | Ahansal Events | https://www.transatlasmarathon.net/ |
| Javelina Jundred | Aravaipa Running | https://aravaiparunning.com/network/javelinajundred/ |
| Desert RATS Kokopelli | Gemini Adventures | https://geminiadventures.com/run/desert-rats-150 |
| Trail Verbier St-Bernard | UTMB | https://verbier.utmb.world/ |
| Grand 2 Grand Ultra | Grand 2 Grand Ultra | https://g2gultra.com/ |
| Fat Dog 120 | Fatdog Management | https://www.fatdog120.ca/ |
| Gorge Waterfalls 50K, 100K | Daybreak Racing | https://www.daybreakracing.com/gorge-waterfalls |
| Squamish 50 | Coast Mountain Trail Running | https://squamish50.com/ |
| Canadian Death Race | Sinister Sports | https://www.sinistersports.ca/ |
| Haliburton Forest Races | Haliburton Forest Trail Ultra | https://www.haliburtonforest100.org/ |
| Hardrock 100 | Hardrock Hundred Board of Directors | https://hardrock100.com/ |
